# Repurposing of the Malaria Box for *Babesia microti* in mice identifies novel active scaffolds against piroplasmosis

**DOI:** 10.1186/s13071-022-05430-4

**Published:** 2022-09-19

**Authors:** Mohamed Abdo Rizk, Hanadi B. Baghdadi, Shimaa Abd El-Salam El-Sayed, Rasha Eltaysh, Ikuo Igarashi

**Affiliations:** 1 National Research Center for Protozoan Diseases, Obihiro University of Agriculture and Veterinary Medicine, Inada-Cho, Obihiro, Hokkaido Japan; 2 Department of Internal Medicine and Infectious Diseases, Faculty of Veterinary Medicine, Mansoura University, Mansoura, Dakahlia Egypt; 3 Biology Department, College of Science, Imam Abdulrahman Bin Faisal University, Dammam, Saudi Arabia; 4 Basic and Applied Scientific Research Center (BASRC), Imam Abdulrahman Bin Faisal University, Dammam, Saudi Arabia; 5 Department of Biochemistry and Chemistry of Nutrition, Faculty of Veterinary Medicine, Mansoura University, Mansoura, Dakahlia Egypt; 6 Department of Pharmacology, Faculty of Veterinary Medicine, Mansoura University, Mansoura, Dakahlia Egypt

**Keywords:** *Babesia microti*, Malaria Box, Bioinformatics analysis, MMV396693, MMV665875

## Abstract

**Background:**

An innovative approach has been introduced for identifying and developing novel potent and safe anti-*Babesia* and anti-*Theileria* agents for the control of animal piroplasmosis. In the present study, we evaluated the inhibitory effects of Malaria Box (MBox) compounds (*n* = 8) against the growth of *Babesia microti* in mice and conducted bioinformatics analysis between the selected hits and the currently used antibabesial drugs, with far-reaching implications for potent combinations.

**Methods:**

A fluorescence assay was used to evaluate the in vivo inhibitory effects of the selected compounds. Bioinformatics analysis was conducted using hierarchical clustering, distance matrix and molecular weight correlation, and PubChem fingerprint. The compounds with in vivo potential efficacy were selected to search for their target in the piroplasm parasites using quantitative PCR (qPCR).

**Results:**

Screening the MBox against the in vivo growth of the *B. microti* parasite enabled the discovery of potent new antipiroplasm drugs, including MMV396693 and MMV665875. Interestingly, statistically significant (*P* < 0.05) downregulation of cysteine protease mRNA levels was observed in MMV665875-treated *Theileria equi *in vitro culture in comparison with untreated cultures. MMV396693/clofazimine and MMV665875/atovaquone (AV) showed maximum structural similarity (MSS) with each other. The distance matrix results indicate promising antibabesial efficacy of combination therapies consisting of either MMV665875 and AV or MMV396693 and imidocarb dipropionate (ID).

**Conclusions:**

Inhibitory and hematology assay results suggest that MMV396693 and MMV665875 are potent antipiroplasm monotherapies. The structural similarity results indicate that MMV665875 and MMV396693 have a similar mode of action as AV and ID, respectively. Our findings demonstrated that MBox compounds provide a promising lead for the development of new antibabesial therapeutic alternatives.

**Graphical Abstract:**

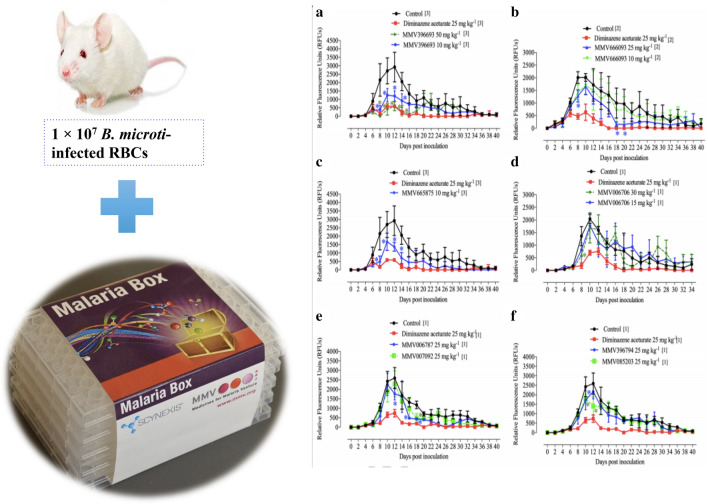

**Supplementary Information:**

The online version contains supplementary material available at 10.1186/s13071-022-05430-4.

## Background

Piroplasmosis is a tick-borne parasitic disease causing great economic loss in the livestock industry worldwide [1]. Diminazene aceturate (DA) and imidocarb dipropionate (ID) have been the standard treatments for babesiosis for many years [2]. However, they have major side effects including a long period for tissue removal, toxicity, and in the case of DA, unavailability in certain countries. Furthermore, new research has revealed that some *Babesia* species such as *Babesia gibsoni* may develop DA resistance [3, 4]. Consequently, searching for potent new antibabesial drugs in the currently available antiprotozoal libraries may help in filling this empty antibabesial drug pipeline. In this regard, we recently conducted a large-scale screening of Malaria Box (MBox) compounds (*n* = 400) against the in vitro growth of *Babesia bovis*, *Babesia bigemina*, *Babesia caballi*, and *Theileria equi*, and 10 potent Medicines for Malaria Venture (MMV) compounds were identified, which showed a wide potential effect against both bovine and equine piroplasm parasites [5, 6]. Since the in vitro effect of these novel candidates does not reveal the effect of host-related factors [7], testing their in vivo inhibitory effect is required. As a result, in this study, the in vivo inhibitory effects of these powerful MMV compounds were investigated against *Babesia microti*, which infects wild small mammals including rodents as the reservoir host [8, 9], and which has served as a useful experimental model for animal babesiosis research [10, 11]. The compounds with potential in vivo efficacy were then selected to investigate their structural similarities with each other, with the commonly used antibabesial drugs (DA, and ID), and with the recently identified antibabesial drug clofazimine (CF), using bioinformatics analysis. Also, the targets of these MMV compounds in the *Babesia* parasite were identified using quantitative polymerase chain reaction (qPCR). Thus, the data presented in this study provide the veterinary field novel compounds with potential efficacy against both bovine babesiosis and equine piroplasmosis. Also, this study suggests a combination therapy for possible use in the treatment of human babesiosis.

## Methods

### In vivo efficacy of MMV compounds and median lethal dose determination

**Fig. 1:**
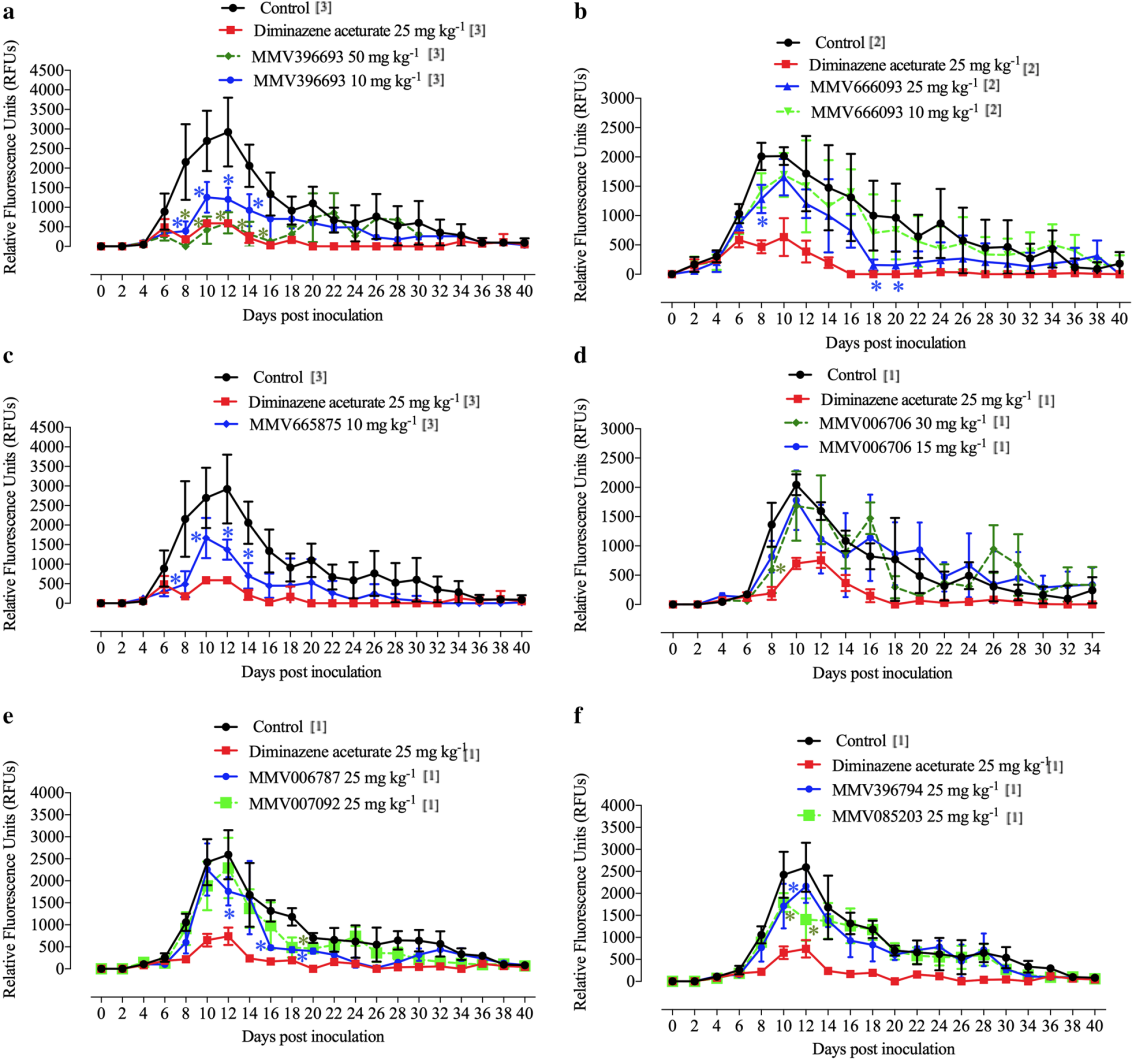
The growth inhibitory effects of MMV compounds with potent in vitro antipiroplasm (bovine *Babesia* and equine *Babesia* and *Theileria*) effects on *B. microti* in BALB/c mice. **a** MMV396693. **b** MMV666093. **c** MMV665875. **d** MMV006706. **e** MMV006787 and MMV007092. **f** MMV396794 and MMV085203. Each value is the mean and SD of the independent experiments. The number of independent experiments is shown in brackets. *P* < 0.05 between treated and untreated mice is indicated by asterisks

The anti-*B. microti* effects of eight MMV compounds (MolPort, Latvia) were investigated in this study in 8-week-old female BALB/c mice (CLEA, Japan) using a fluorescence-based SYBR Green I (SGI) (Lonza, USA; 10,000×) assay [10]. Five mice were allocated to each group. All mice were intraperitoneally injected with 1 × 10^7^ *B. microti* (Munich strain)-infected red blood cells (RBCs), except for one group that remained uninfected to use as a negative control. The level of parasitemia in the infected mice was monitored daily using Giemsa-stained thin blood smears prepared from venous tail blood, and beginning day 4 post-infection (p.i.), when the parasitemia reached approximately 1%, either MMV compounds or control drug (DA, Novartis, Japan) was intraperitoneally administered to the mice for five successive days [10–14]. Double-distilled water, sterile phosphate-buffered saline 1× (PBS 1×), or a sterile PBS 1× and DMSO (dimethylsulfoxide) solution was used for dissolving MMV compounds, with a final concentration of 0.01% to 0.02%, while DA was dissolved in autoclaved physiological saline (0.9% NaCl w/v, pH 7.2) at a dosage rate of 25 mg kg^−1^. Various doses of the selected MMV compounds were administered (Fig. 1). After the start of treatment, the emitted fluorescence signals were monitored every 2 days until the cessation of parasitemia. Next, the median lethal dose (LD_50_) value for MMV396693 was determined using a “staircase” method with doses in ascending order ranging from 10 mg kg^−1^ to 100 mg kg^−1^ (five mice per dose) following a protocol previously described by Randhawa et al. [15]. The staircase technique is a limit method in which stimuli are presented in ascending and descending order. The direction of the stimulus sequence is reversed when the observer’s response changes. Because it does not deliver stimuli that are far above or below the threshold, this strategy is effective [15].

### Hemolytic anemia monitoring

Hematological parameters, including hemoglobin (HGB) levels, RBC counts, and hematocrit (HCT), were monitored every 96 h using a Celltac Alpha MEK-6450 automatic hematology analyzer to assess the efficacy of MMV monotherapy in preventing the progression of anemia in the treated mice as detailed previously [10, 11].

### Atom pair fingerprint measurements

DA, ID, CF, and AV atom pair fingerprints (APfp) were utilized to calculate the structural similarity between the MMV compounds with potent anti-*B. microti* activity (MMV396693 and MMV665875), either with each other or with other antibabesial drugs [16]. The compound identification number (CID) acquired from PubChem for each compound was used to compute the APfp of the two MMV compounds and other antibabesial medicines. The CIDs were entered into ChemMine Web Tools software, which calculated the APfp of all compounds [17]. ChemmineR software was used to analyze the APfp for hierarchical cluster analysis (HCA) [18].

### RNA extraction and qPCR

To evaluate the effects of treatment with the potent identified antipiroplasm MMV compound MMV665875 on the messenger RNA (mRNA) level of the target gene, qPCR was used. *Babesia bovis* (Texas strain) [19, 20] and *T. equi* (United States Department of Agriculture [USDA] strain) [6, 21] were cultivated in 24-well plates using a microaerophilic, stationary-phase culture system [19, 22] and treated with the IC_99_ (is approximately 100-fold the IC_50_ concentration) of the specific potent MMV compound for 8 h. A culture that received no treatment was used as a negative control. RBCs were collected from all cultures and washed with PBS. Then, total RNA was extracted, complementary DNA (cDNA) was synthesized, and the SGI PCR Master Mix (Lonza, USA) was used as detailed in our previous study [23]. Target gene fold alterations in comparison with *18S rRNA* [24, 25] were estimated in MMV665875-treated and DMSO-treated *B. bovis* or *T. equi* as described previously [26].

The assay was used for amplification of the cysteine protease (CP) (GenBank accession numbers AK441400 for *B. bovis* [CP2] and XM_004833324.1 for *T. equi*) gene from RNA extracted from either MMV-treated or DMSO-treated (control) cultures. Specific forward and reverse primers were designed by Primer3Plus software (http://www.bioinformatics.nl/cgi-bin/primer3plus/primer3plus.cgi/) and used to amplify the target genes (Additional file 3: Table S3).

### Statistical analysis

The obtained data were analyzed using GraphPad Prism. Differences between the control and treated groups were determined by one-way analysis of variance (ANOVA) and unpaired *t*-tests [11, 27]. A *P*-value < 0.05 was considered statistically significant.

## Results

### Effects of MBox compounds on *B. microti* growth in mice

The MBox richness as a resource for creating strong, new antipiroplasm agents was discovered through the in vitro screening of 400 MMV compounds from this box against the growth of *B. bovis, B. bigemina*, *T. equi*, and *B. caballi* parasites [5, 6]. An in vivo investigation was conducted utilizing *B. microti* in a mouse model to further confirm the MMV compounds as antipiroplasm agents. The highest fluorescence values in the positive control group ranged from 2016 to 2922 (Fig. 1). When compared with control mice, 50 mg kg^−1^ and 10 mg kg^−1^ MMV396693 significantly reduced fluorescence levels (*P* < 0.05) (ANOVA: *F*_(1.121, 22.56)_ = 17.33, *P* = 0.0002) from days 8 to 16 and from days 8 to 14 p.i., respectively (Fig. 1a). Fluorescence values were significantly lower (ANOVA: *F*_(1.479, 29.58)_ = 28.09, *P* < 0.0001) in mice treated with 25 mg kg^−1^ MMV666093 at days 8, 18, and 20 p.i. as compared with those in the controls (Fig. 1b), while treatment with 10 mg kg^−1^ MMV665875 exhibited significant inhibition (ANOVA: *F*_(1.101, 22.03)_ = 19.81, *P* = 0.0001) of fluorescence signals from day 8 to day 14 p.i. as compared with that in the controls (Fig. 1c). Administration of 30 mg kg^−1^ MMV006706 led to significant inhibition (ANOVA: *F*_(2.593, 44.09)_ = 12.99, *P* < 0.0001) of the emitted fluorescence signals at day 8 p.i. in comparison with *B. microti*-infected untreated mice (Fig. 1d).

**Fig. 2:**
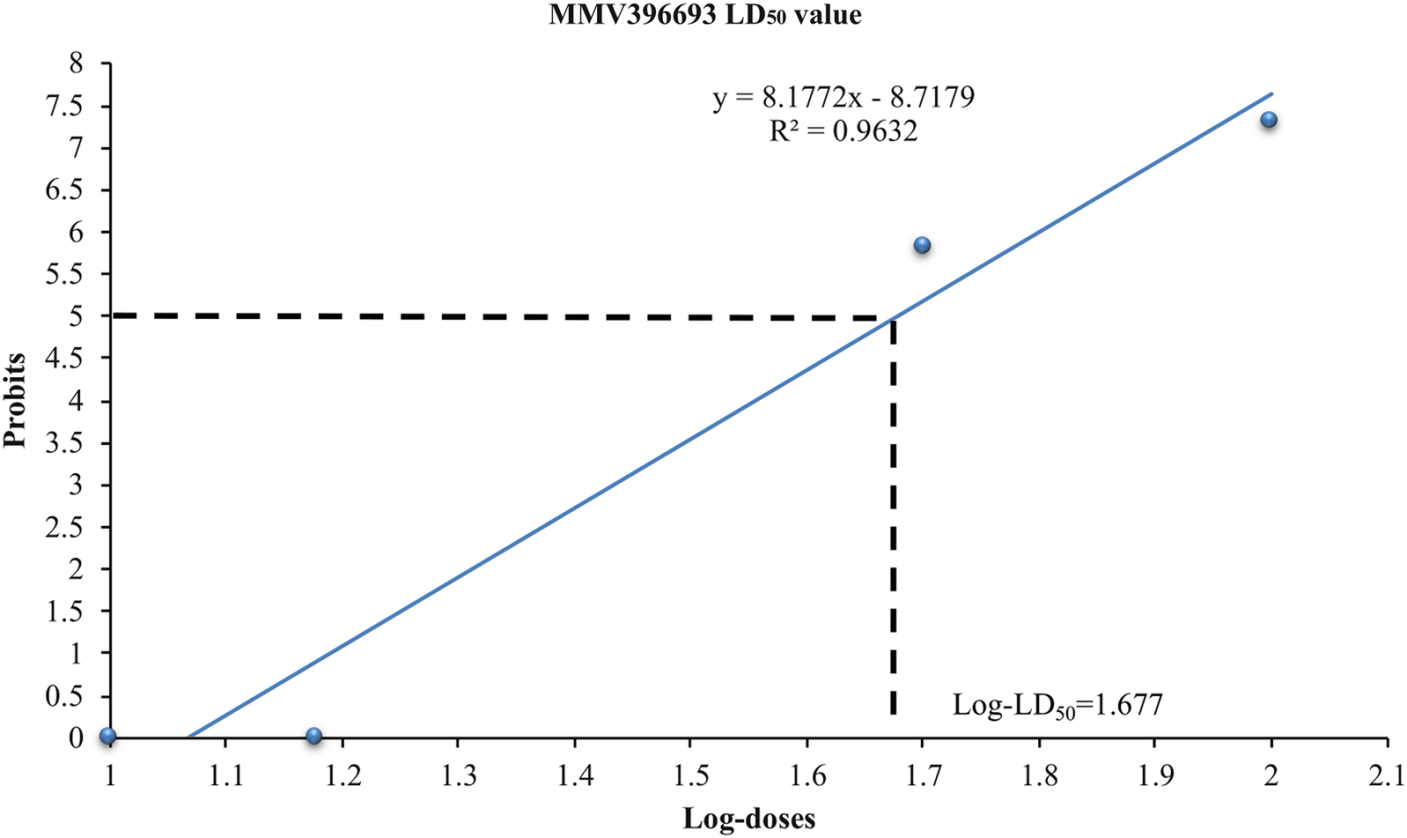
A plot of log doses versus probits for calculation of the LD_50_ of MMV396693 administered intraperitoneally. The experiment was repeated twice

Inhibition percentages in the in vivo growth of *B. microti* in the area under the curve for all tested drugs are shown in Additional file 3: Table S1. Collectively, 10 mg kg^−1^ MMV665875 exhibited the highest anti-*B. microti* efficacy in vivo among the screened MMV compounds, followed by 10 mg kg^−1^ MMV396693 and 25 mg kg^−1^ MMV666093 (Additional file 3: Table S1). Treatment with 25 mg kg^−1^ MMV007092, MMV006787, MMV396794, and MMV085203 resulted in inhibition of 11.64%, 32.01%, 16.59%, and 45.77% at day 12 p.i., respectively (Fig. 1e, f). Treatment with 10 mg kg^−1^ and 50 mg kg^−1^ MMV396693 achieved 53.44% and 84.53% inhibition of *B. microti* growth at day 10 p.i. and 58.78% and 79.36% inhibition at day 12 p.i., respectively. The inhibition rates after treatment with 25 mg kg^−1^ DA were 78.05% and 79.75% at days 10 and 12 p.i., respectively (Fig. 1a). However, the inhibitory effect of 50 mg kg^−1^ MMV396693 caused red discoloration of the mice’s urine, with signs of toxicity such as loss of body weight observed in the treated mice at day 8 p.i. Then the mice started to die, and at day 30 p.i., only two mice remained in this treated group. In light of this issue, we ended the experiment for this dose (50 mg kg) ^−1^at day 30 p.i. and performed a pilot study to calculate the LD_50_ value for MMV396693. The result revealed that the LD_50_ for MMV396693, when administered intraperitoneally in mice, was 47.59 mg kg^−1^ (Fig. 2).

Eight days p.i., the rates of *B. microti* growth inhibition in mice caused by 10 mg kg^−1^ and 25 mg kg^−1^ MMV666093 were 28.89% and 36.21%, respectively (Fig. 1b). Treatment with 30 mg kg^−1^ MMV006706 caused 17.83% inhibition in the growth of *B. microti* in mice (Fig. 1d). Interestingly, 10 mg kg^−1^ MMV665875 caused 38.05% and 53.04% inhibition in the growth of *B. microti* in mice at days 10 and 12 p.i., respectively (Fig. 1c). These results highlight the potential antipiroplasm efficacy of MMV396693 and MMV665875 (both probe-like compounds) when used as monotherapies.

### Effect of MBox compounds on anemia

**Fig. 3:**
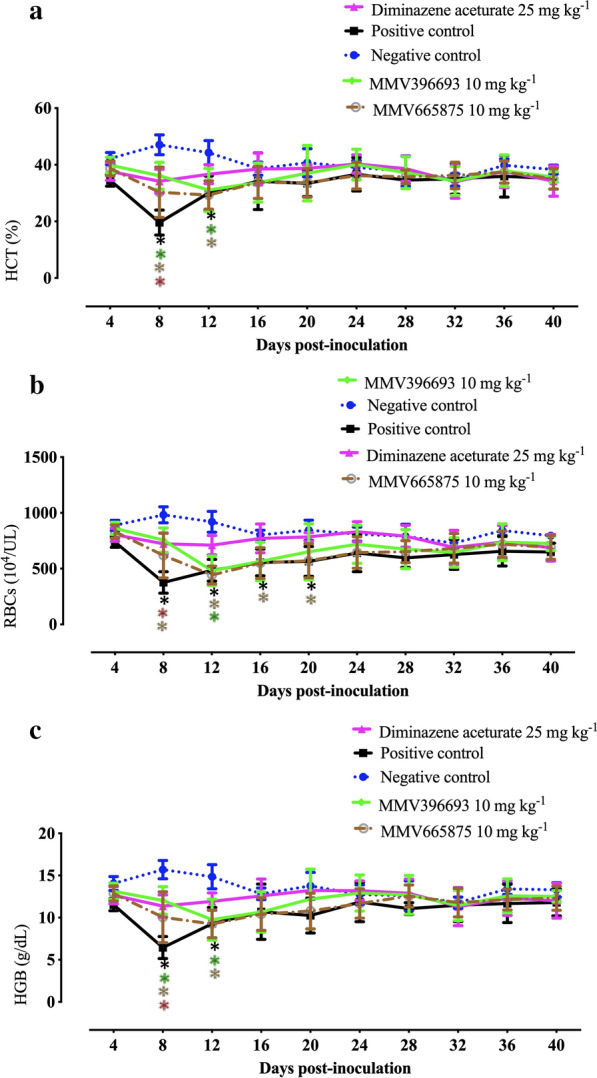
Hematological variables in MBox compound-treated BALB/c mice. **a** Hematocrit (HCT) levels. **b** Red blood cell (RBC) counts. **d** Hemoglobin (HGB) values. Each value is represented as mean and SD of three separate experiments. *P* < 0.05 between treated and untreated mice is indicated by asterisks

In this study, HGB levels, RBC counts, and HCT values were calculated to track the recovery of mice from hemolytic anemia caused by *B. microti* after treatment with powerful MMV antipiroplasm drugs. HGB levels and HCT values were restored to normal levels at day 16 p.i. after treatment with 10 mg kg^−1^ MMV665875 (Fig. 3a, c). Treatment with MMV396693 (10 mg kg^−1^) demonstrated better results than that with MMV665875, as the former restored normal RBC counts at day 16 p.i., whereas reduced RBC counts were detected in MMV665875-treated mice until day 20 p.i. (Fig. 3b). These data highlight the quick recovery from anemia caused by *B. microti* infection once treatment with MMV396693 is initiated and confirm the promising antibabesial efficacy of this hit. Unfortunately, intraperitoneal treatment with 50 mg kg^−1^ MMV396693 caused significant reductions (ANOVA: *F*_(1.577, 9.461)_ = 11.73, *P* = 0.0038 for HGB, ANOVA: *F*_(1.808, 10.98)_ = 12.95, *P* = 0.0016 for HCT, and ANOVA: *F*_(1.534, 9.207)_ = 12.05, *P* = 0.0039 for RBCs) in all hematological parameters from day 8 p.i. until day 28 p.i. in comparison with negative control mice (Additional file 1: Fig. S1).

### Maximum structural similarity between MBox compounds and antibabesial drugs

**Fig. 4:**
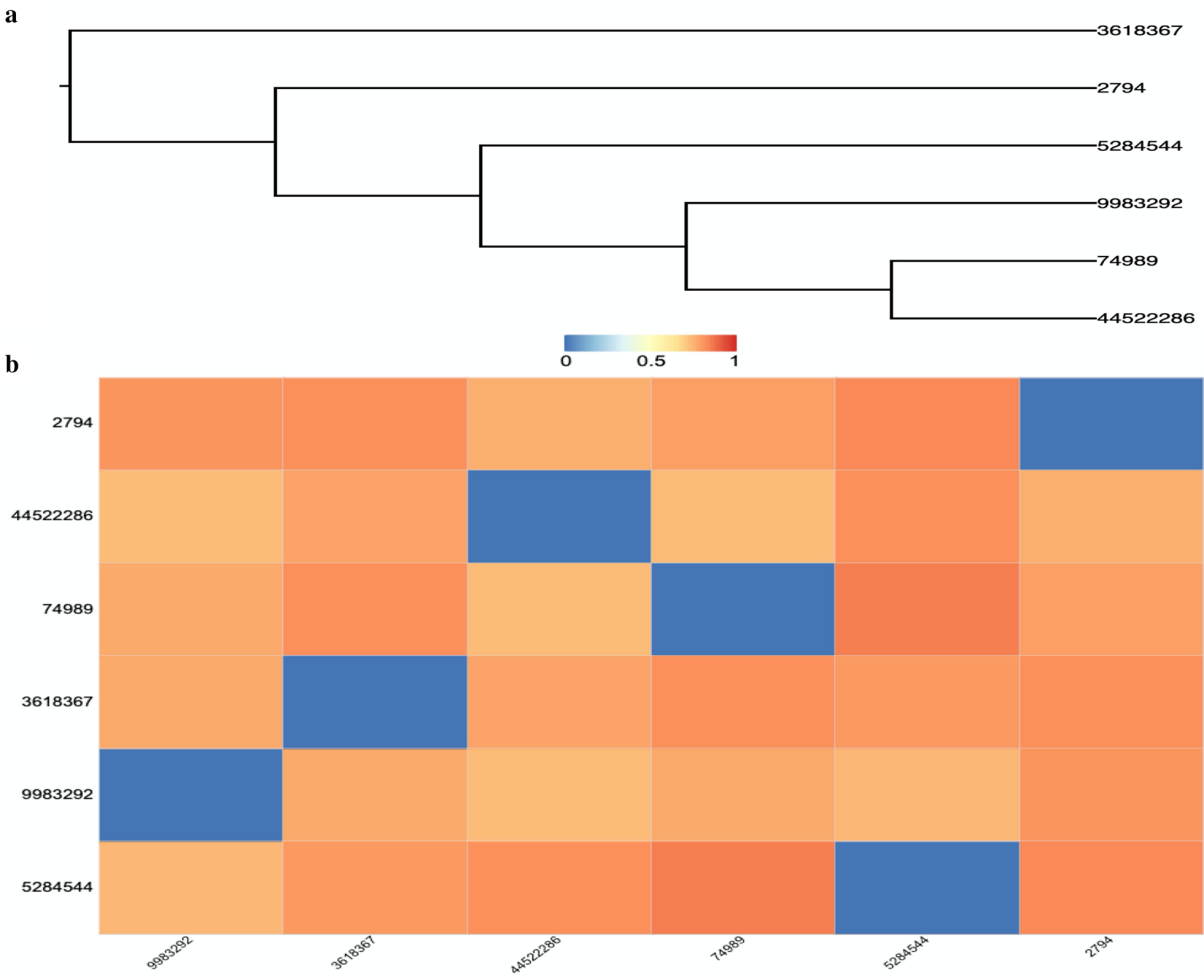
Measurements of structural similarity. **a** Analysis of hierarchical clustering. ChemmineR was used to perform the hierarchical cluster analysis. **b** A heatmap depicting the distance matrix between diminazene aceturate, imidocarb dipropionate, clofazimine, and atovaquone, which were identified as effective MMV compounds. With Z-score display values, a single-linkage mechanism was used. 3618367 = MMV396693, 44522286 = MMV665875, 5284544 = diminazene aceturate, 9983292 = imidocarb dipropionate, 2794 = clofazimine, and 74989 = atovaquone

MMV396693 (CID: 3618367) exhibited the maximum structural similarity (MSS) to CF (CID: 2794) in the HCA (Fig. 4a). MSS was found between CF (CID: 2794), DA (CID: 5284544), and ID (CID: 9983292) in the same fashion (Fig. 4a). Based on this analysis, AV (CID: 74,989) and MMV665875 (CID: 44522286) both demonstrated MSS with each other (Fig. 4a). Compounds that fall into different clusters have structural differences from one another and from other antibabesial medicines, indicating a possible alternative antibabesial mechanism.

### Distance matrix between MBox compounds and antibabesial drugs

With both AV and ID, MMV665875 exhibited the lowest distance matrix (LDM) (Fig. 4b and Additional file 3: Table S2). ID showed the LDM with MMV396693 (CID: 3618367) (Fig. 4b and Additional file 3: Table S2). MMV396693 (CID: 3618367) and MMV665875 (CID: 44522286) exhibited very similar molecular weight as that of CF (CID: 2794) (Additional file 2: Fig. S2). Similarity workbench results showed 0.26 AP Tanimoto values between either MMV665875 and AV or MMV665875 and ID, with maximum common substructure (MCS) values of 9 and 10, respectively (Table 1). AP Tanimoto values, which measure the overlap between descriptors for two compounds and are calculated as the ratio between conserved features and the total number of features of each molecule, were relatively high (0.22) between MMV396693 and ID and between MMV665875 and CF (Table 1). Collectively, distance matrix correlation and fingerprint for similarity workbench results highlight the possible potential antibabesial efficacy of either MMV665875 and AV or MMV396693 and ID when administered as a combination therapy.

**Table 1:** Similarity workbench between the identified potent MMV compounds and the commonly used and recently identified antibabesial drugs

	MMV396693 & MMV665875	MMV396693 & AV	MMV396693 & DA	MMV396693 & ID	MMV396693 & CF	MMV665875 & AV	MMV665875 & DA	MMV665875 & ID	MMV665875 & CF
AP Tanimoto	0.21	0.17	0.19	0.22	0.16	0.26	0.16	0.26	0.22
MCS Tanimoto	0.17	0.15	0.14	0.17	0.18	0.20	0.13	0.18	0.21
MCS size	7	6	7	8	8	9	8	10	11

*AV* atovaquone, *DA* diminazene aceturate, *ID* imidocarb dipropionate, *CF* clofazimine, *MCS* maximum common substructure

### MMV665875 downregulates mRNA of the cysteine protease gene in *T. equi*

**Fig. 5:**
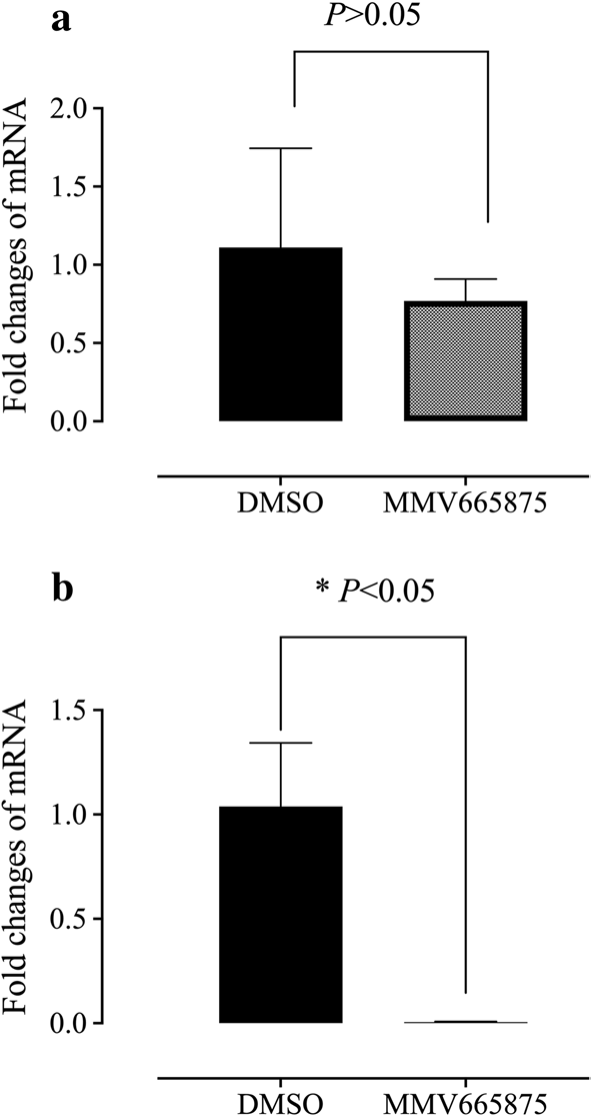
mRNA level of the cysteine protease (CP) gene from *B. bovis* and *T. equi* cultures treated with MMV665875 at their IC_99_ values and DMSO (0.1%) for 8 h using qPCR. **a** *B. bovis*. **b** *T. equi*. *P* < 0.05 between the fold changes is indicated by asterisks. Three independent experiments were performed

qPCR was utilized to look for probable targets of the newly identified powerful MMV compounds in *Babesia* parasites. The in vitro treatment of *B. bovis* with the IC_99_ of MMV665875 for 8 h downregulated the CP2 gene (Fig. 5a). Of note, no statistically significant difference (ANOVA: *F*_(1, 1)_ = 11.73, *P* = 0.4121) was observed in the effects on the CP2 gene in the MMV665875-treated parasites as compared with the control (Fig. 5). Interestingly, a statistically significant difference (ANOVA: *F*_(1, 1)_ = 322.5, *P* = 0.0042) was observed in the CP (Fig. 5b) mRNA levels in MMV665875-treated *T. equi*, with substantial downregulation in comparison with DMSO-treated cultures. The results showed that CP could serve as a target gene of MMV665875 for parasite inhibition in *T. equi*.

### Discussion

The MBox was created to provide a novel avenue for identifying agents to treat apicomplexan parasites [28]. The in vitro inhibitory effects of MBox compounds against 16 protozoa were published in a large, comprehensive dataset [5]. However, the in vivo inhibitory effects, pharmacokinetic/dynamic properties, and LD_50_ values of most MMV compounds are still unknown. Our recently published in vitro results [5, 6] revealed that 17, 2, and 10 MMV hits were the most interesting with regard to bovine *Babesia* parasites (*B. bovis* and *B. bigemina*), equine *Babesia* and *Theileria* parasites (*T. equi* and *B. caballi*), and both bovine and equine *Babesia* and *Theileria* parasites (*B. bovis*, *B. bigemina, T. equi*, and *B. caballi*), respectively. We recently evaluated the in vivo anti-*B. microti* effects of the hits with respect to potent anti-bovine *Babesia* effects (*n* = 17) [29] and anti-equine piroplasm effects (*n* = 2) [23]. However, the in vivo inhibitory effects of the hits demonstrating wide potential in vitro effects against both bovine and equine piroplasm parasites (*B. bovis*, *B. bigemina*, *T. equi* and *B. caballi*) remained to be evaluated. Therefore, in the present study, we evaluated the inhibitory effects of these hits against the growth of *B. microti* in mice. Compounds with potential in vitro antipiroplasm efficacy were chosen based on three primary criteria: (i) compounds with the highest selectivity indices, (ii) those with the lowest IC_50_ values, and (iii) those that are easiest to obtain. Out of 10 compounds with potent antipiroplasm activity, MMV396693, MMV666093, MMV006706, and MMV665875 were chosen because they had the lowest IC_50_ values and the highest selectivity index values. Only MMV085203, MMV396794, MMV006787, and MMV007092 were included in the current in vivo investigation among the remaining MMV compounds (*n* = 6) due to their availability for commercial purchase.

Out of eight MMV compounds evaluated against the growth of *B. microti* in mice, MMV396693 and MMV665875 showed potential in vivo activity when used as monotherapy. Treatment of the mice with these two MMV compounds demonstrated that 10 mg kg^−1^ MMV396693, and 10 mg kg^−1^ MMV665875 resulted in 81.65% and 77.35% inhibition, respectively, at day 8 p.i. These results are higher than the 21% inhibition for 100 mg kg^−1^ enoxacin, 15% for 150 mg kg^−1^ norfloxacin, 23% for 700 mg kg^−1^ ofloxacin [11], 26% for 125 mg kg^−1^ pyronaridine tetraphosphate [10], 37% for 50 mg kg^−1^ thymoquinone [27], 40.38% for 130 mg kg^−1^ *Zingiber officinale* rhizome [30], and 65.57% for 200 mg kg^−1^ myrrh oil [12] monotherapies. On the contrary, the inhibition of *B. microti* growth in mice from MMV396693 was lower than the 90% inhibition for tafenoquine [31].

Van Voorhis et al. (2016) demonstrated that the CP gene may be the target for MMV665875 to inhibit the growth of Apicomplexa parasites such as *Plasmodium falciparum *in vitro, while the protozoan target of MMV396693 is still unknown.

Indeed, *B. microti* and other bovine and equine *Babesia/Theileria* parasites share biological and parasitological characteristics; for example, all of these parasites are transmitted by ticks, invade RBCs and replicate within them, and cause similar clinical symptoms in dogs, cattle, and equines. Therefore, we evaluated the effects of treatment with the identified antipiroplasm MMV compound on the mRNA level of the target gene in *B. bovis* and *T. equi* parasites using qPCR. The IC_99_ of MMV665875 significantly downregulated the mRNA of the CP gene in the *T. equi* culture (*P* < 0.05), indicating that this gene may be the target of this probe-like compound in *T. equi*. CPs are present in all living organisms and play a critical role in the host cell invasion of many protozoan parasites [32, 33]. For *Plasmodium*, CPs play a key role in the egression process through their involvement in the degradation of both HGB and erythrocyte cytoskeletal proteins, with the subsequent rupture of the infected erythrocytes [33]. Therefore, CPs represent promising new drug targets for several protozoan diseases, including trypanosomiasis [34], malaria [32], schistosomiasis [35], and leishmaniasis [36]. For piroplasm parasites, a previous in vitro study [37] suggested the presence of CPs in *B. bovis.* As a result of progress in genomic analysis [38], a subsequent study confirmed the presence of CPs in *Babesia* and characterized CP in *B. bovis*. Okubo et al. [37] proposed an essential role of CPs in the invasion of *B. bovis* to the host RBCs. Ascencio et al. [39] reported that *T. equi* exhibited extreme expansion of C1A-CP paralogs, which might be functionally associated with the evolution of the schizont stage. However, the exact function of babesial CPs remains uncertain. Although the present study revealed significant downregulation in the mRNA of the CP gene in the *T. equi* culture treated with MMV665875, more in-depth studies are needed to confirm this possible identified target. Also, further studies are essential to identify the target genes of MMV665875 in *B. microti*.

Generally speaking, DA and ID are the current standard treatment for animal babesiosis, and over time, antibabesial drug resistance has emerged, especially against DA [3, 4]. Furthermore, the administration of ID is commonly associated with adverse effects, including pain during injection and mild cholinergic signs such as salivation, nasal drip, and brief episodes of vomiting [2]. Simultaneous administration of atropine sulfate is required to reverse the associated cholinergic signs [2]. For human babesiosis, combination therapies are the current regime for treating the infection, with reporting of recovery failure in severe cases and the development of drug-resistant parasites [40]. Therefore, the development of novel combination therapies consisting of low doses of the common animal or human antibabesial drugs with the novel identified MMV compounds might be an effective alternative strategy. Following this pattern, in this study, structural similarities of these MMV compounds either with each other or with the common antibabesial drugs were determined using bioinformatics analysis. In cheminformatics, quantifying the similarity of two molecules is a crucial concept and a common task [41]. Its applications span a variety of domains, the majority of which are connected to medicinal chemistry, such as virtual screening [42]. HCA in the current study revealed MSS between MMV396693 and CF. When the combination was administered to *Babesia*-infected animal models, the molecular weight correlation heatmap supported the potential promising antibabesial efficacy. Similarly, the distance matrix correlation, similarity workbench fingerprint, and HCA revealed the potential antibabesial efficacy of both MMV665875/AV and MMV396693/ID when administered as combination therapy. Future research is needed to look into the synergistic interactions of these combination therapies against *Babesia* parasites in vitro and in *B. microti*-infected mice.

## Conclusions

Potent antipiroplasm drugs MMV396693 and MMV665875 were identified in the present study. In vitro treatment of *T. equi* with the IC_99_ of MMV665875 for 8 h significantly downregulated the mRNA levels of the CP gene. MMV396693/CF and MMV665875/AV both showed MSS. The distance matrix and similarity workbench fingerprint results highlight the fact that MMV665875 and MMV396693 may have similar modes of action as AV and ID, respectively. This treatment could be used to treat babesiosis in both animals and humans. The targets and mechanisms of action of these drugs may bring novel insights into the biology of *Babesia* and *Theileria.*

## Supplementary Information

**Additional file 1: Figure S1. **Anemia monitoring in *B. microti*-infected mice treated with 50 mg kg^−1^ MMV396693. **a** RBC counts. **b** HGB levels. **c** Hematocrit values. Each value is the mean and SD of the independent experiments. Asterisks indicate statistically significant (*P < 0.05) difference between treated and untreated mice.

**Additional file 2: Figure S2.** The molecular weight association between the powerful MMV drugs diminazene aceturate, imidocarb dipropionate, clofazimine, and atovaquone is shown in a heatmap. With Z-scores display values, a single-linkage mechanism was used. 3618367 = MMV396693, 44522286 = MMV665875, 5284544 = diminazene aceturate, 9983292 = imidocarb dipropionate, 2794 = clofazimine, and 74989 = atovaquone.

**Additional file 3: Table S1.** Inhibitory effects of the tested MMV compounds with potential against the growth of *B. microti* in mice in comparison with a positive control group. **Table S2.** Distance matrix correlation between MBox compounds with potential and the currently used antibabesial drugs [diminazene aceturate (DA), imidocarb dipropionate (ID), clofazimine (CF), and atovaquone (AV)]. **Table S3.** Primers used for determining the mRNA level of the expected target gene from *B. bovis* and *T. equi* cultures treated with MMV665875 at their IC_99_ values and DMSO (0.1%) used for 8 h using a qPCR.

## Data Availability

The datasets generated during and/or analyzed during the current study are available from the corresponding author on reasonable request.

## Abbreviations

MBox: Malaria Box
*B. microti*: *Babesia microti*
qPCR: quantitative PCR
MSS: Maximum structural similarity
AV: Atovaquone
ID: Imidocarb dipropionate
DA: Diminazene aceturate
*B. bovis*: *Babesia bovis*
*T. equi*: *Theileria equi*
MMV: Medicines for Malaria Venture
CF: Clofazimine
SGI: SYBR Green I
DMSO: Dimethylsulfoxide
HGB: Hemoglobin
RBC: Red blood cell
HCT: Hematocrit
APfp: Atom pair fingerprint
CID: Compound identification number
HCA: Hierarchical cluster analysis
USDA: United States Department of Agriculture
CP: Cysteine protease
