# Supplementary figures and images for: Repurposing of the Malaria Box for Babesia microti in mice identifies novel active scaffolds against piroplasmosis
Source: Parasit Vectors. 2022 Sep 19;15:329. doi: 10.1186/s13071-022-05430-4 (PMC9487043; doi:10.1186/s13071-022-05430-4)

A.

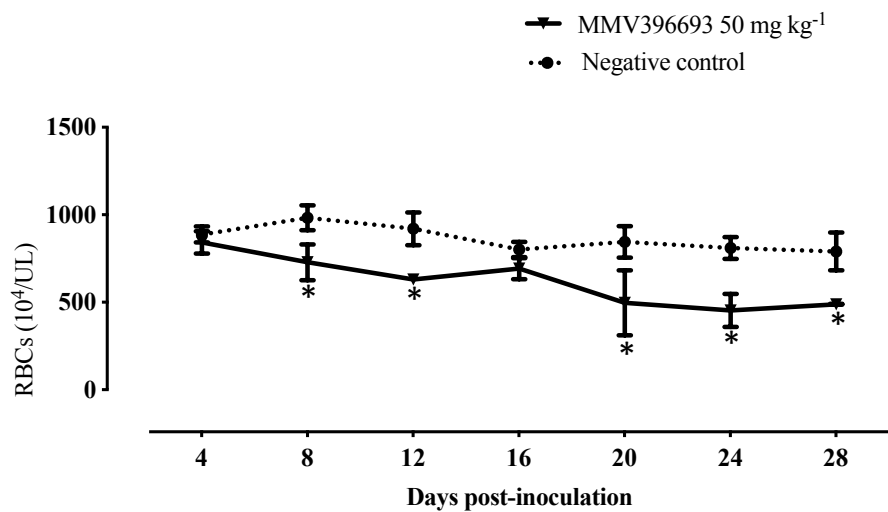

B.

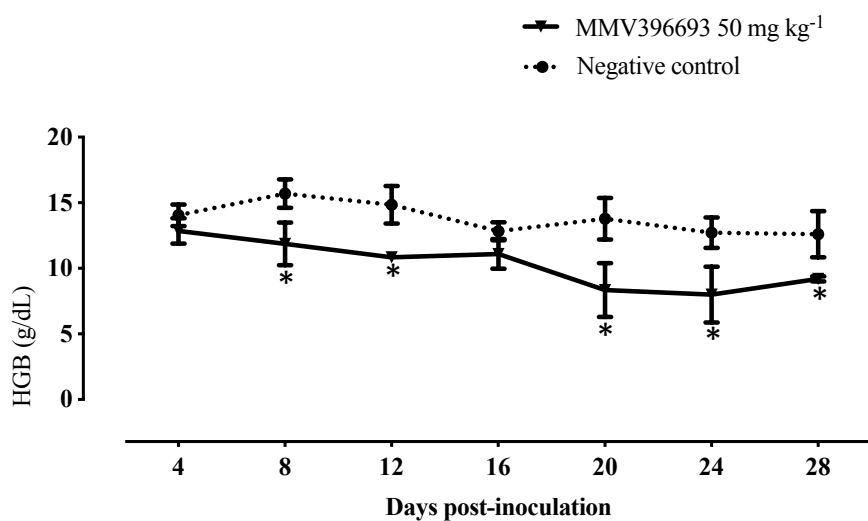

C.

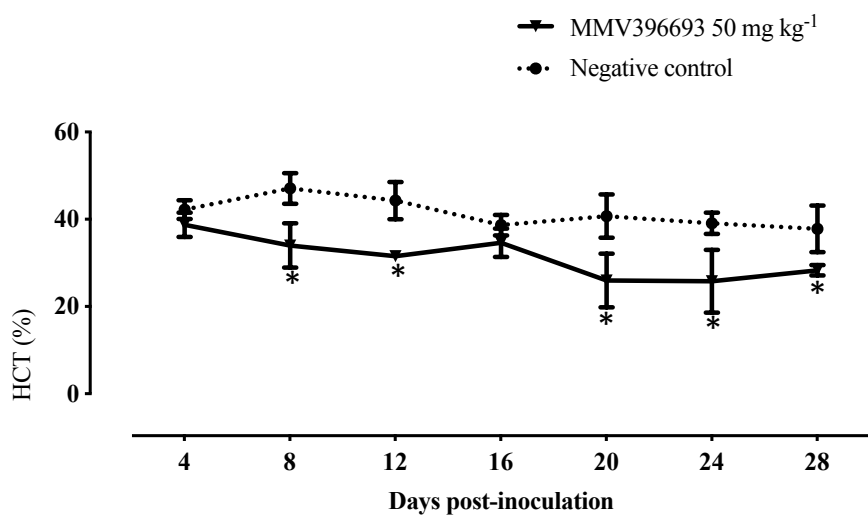

Supplement: Supplementary file 1 — Additional file 1: Figure S1. Anemia monitoring in B. microti-infected mice treated with 50 mg kg−1 MMV396693. a RBC counts. b HGB levels. c Hematocrit values. Each value is the mean and SD of the independent experiments. Asterisks indicate statistically significant (*P < 0.05) difference between treated and untreated mice. [file 13071_2022_5430_MOESM1_ESM.pdf]

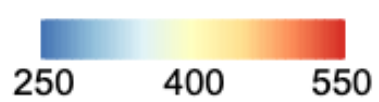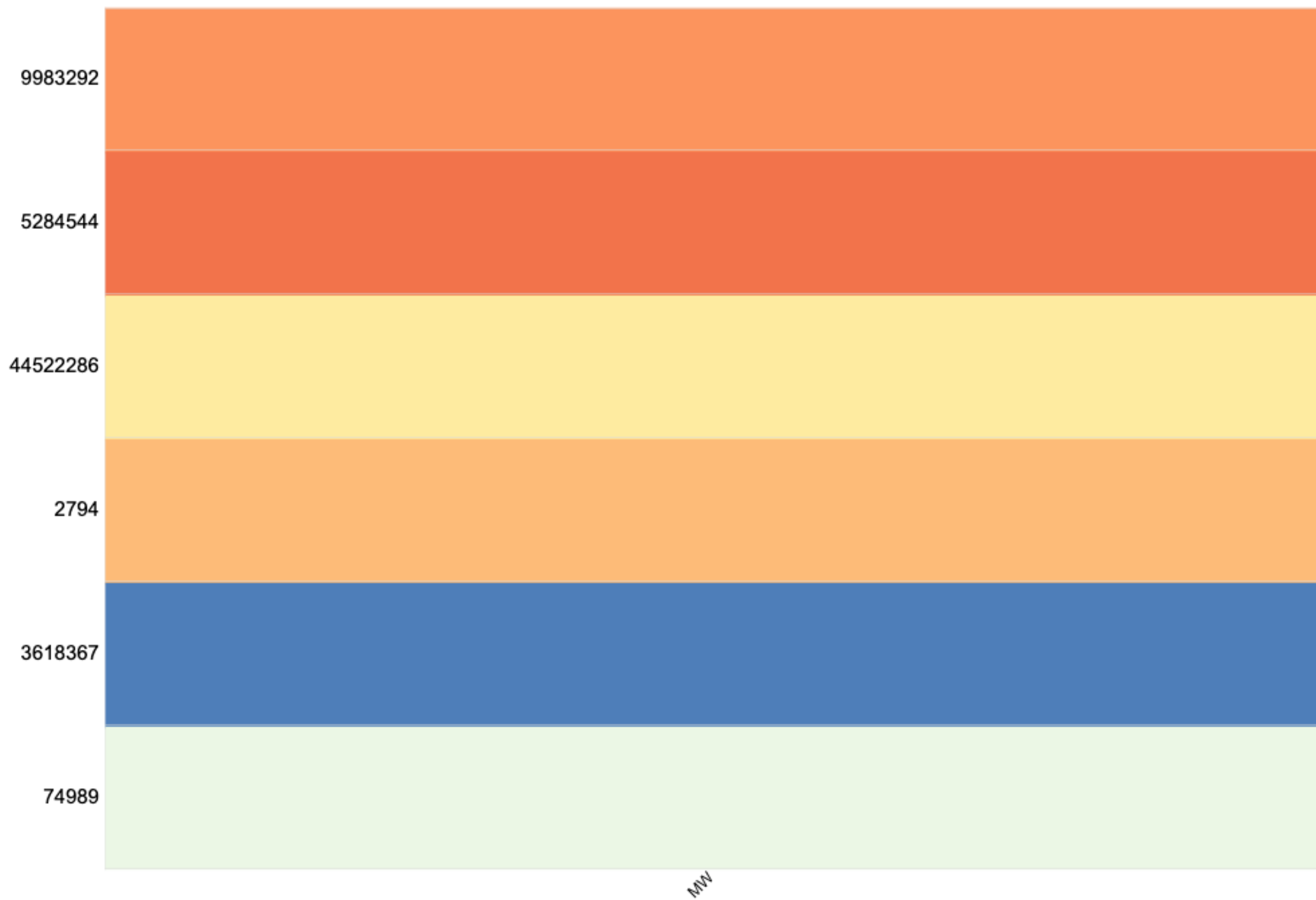

Supplement: Supplementary file 2 — Additional file 2: Figure S2. The molecular weight association between the powerful MMV drugs diminazene aceturate, imidocarb dipropionate, clofazimine, and atovaquone is shown in a heatmap. With Z-scores display values, a single-linkage mechanism was used. 3618367 = MMV396693, 44522286 = MMV665875, 5284544 = diminazene aceturate, 9983292 = imidocarb dipropionate, 2794 = clofazimine, and 74989 = atovaquone. [file 13071_2022_5430_MOESM2_ESM.pdf]
