# Supplementary material for: Repurposing of the Malaria Box for Babesia microti in mice identifies novel active scaffolds against piroplasmosis
Source: Parasit Vectors. 2022 Sep 19;15:329. doi: 10.1186/s13071-022-05430-4 (PMC9487043; doi:10.1186/s13071-022-05430-4)
Supplement: Supplementary file 3 — Additional file 3: Table S1. Inhibitory effects of the tested MMV compounds with potential against the growth of B. microti in mice in comparison with a positive control group. Table S2. Distance matrix correlation between MBox compounds with potential and the currently used antibabesial drugs [diminazene aceturate (DA), imidocarb dipropionate (ID), clofazimine (CF), and atovaquone (AV)]. Table S3. Primers used for determining the mRNA level of the expected target gene from B. bovis and T. equi cultures treated with MMV665875 at their IC99 values and DMSO (0.1%) used for 8 h using a qPCR. [file 13071_2022_5430_MOESM3_ESM.docx]

| Days post inoculation | MMV396794 25 mg kg^-1^ | MMV085203 25 mg kg^-1^ | MMV006787 25 mg kg^-1^ | MMV007092 25 mg kg^-1^ | MMV396693 10 mg/kg | MMV665875 10mg/kg | MMV006706 30 mg/kg | MMV666093 25 mg kg^-1^ |
| --- | --- | --- | --- | --- | --- | --- | --- | --- |
| 0 | 0.00 | 0.00 | 0.00 | 0.00 | 0.00 | 0.00 | 0.00 | 0.00 |
| 2 | 0.00 | 0.00 | 0.00 | 0.00 | 0.00 | 0.00 | 0.00 | 63.74 |
| 4 | 0.00 | 33.99 | 4.59 | 0.00 | 0.00 | 0.00 | 0.00 | 31.64 |
| 6 | 40.76 | 29.01 | 59.81 | 50.27 | 61.93 | 66.62 | 64.96 | 16.70 |
| 8 | 27.37 | 15.83 | 43.24 | 13.42 | 81.65 | 77.35 | 56.95 | 36.21 |
| 10 | 29.27 | 26.02 | 6.87 | 22.21 | 53.44 | 38.06 | 17.83 | 17.92 |
| 12 | 16.60 | 45.78 | 32.02 | 11.64 | 58.79 | 53.04 | 0.00 | 29.90 |
| 14 | 17.80 | 18.24 | 3.73 | 18.45 | 55.07 | 65.77 | 17.52 | 32.37 |
| 16 | 29.58 | 4.08 | 63.44 | 25.55 | 47.55 | 66.33 | 0.00 | 43.27 |
| 18 | 29.87 | 0.17 | 63.15 | 58.73 | 23.28 | 50.71 | 61.63 | 84.13 |
| 20 | 13.11 | 2.34 | 42.23 | 34.14 | 45.62 | 51.62 | 69.81 | 84.27 |
| 22 | 0.00 | 10.93 | 51.29 | 17.58 | 27.45 | 60.66 | 0.00 | 69.33 |
| 24 | 0.00 | 10.48 | 76.75 | 0.00 | 16.87 | 84.26 | 37.42 | 72.04 |
| 26 | 15.95 | 0.28 | 95.18 | 34.17 | 69.62 | 67.48 | 0.00 | 53.73 |
| 28 | 0.00 | 6.62 | 76.92 | 45.99 | 66.70 | 79.16 | 0.00 | 53.10 |
| 30 | 47.85 | 49.57 | 49.78 | 66.60 | 57.20 | 88.85 | 0.00 | 60.75 |
| 34 | 65.69 | 56.72 | 22.27 | 71.67 | 27.60 | 100.00 | 0.00 | 51.63 |
| 36 | 68.27 | 68.27 | 3.14 | 62.71 | 9.49 | 100.00 | 0.00 | 57.21 |
| 38 | 10.11 | 0.00 | 20.97 | 68.27 | 0.00 | 100.00 | ND | 0.00 |
| 40 | 15.45 | 27.94 | 0.00 | 0.00 | 15.77 | 100.00 | ND | 0.00 |

Supplementary data:

Table S1. Inhibitory effects of the tested MMV compounds with potential against the growth of *B. microti* in mice in comparison with positive control group

Table S2. Distance matrix correlation between MBox compounds with potential and, the currently used antibabesial drugs (diminazene aceturate (DA), imidocarb dipropionate (ID), clofazimine (CF), and atovaquone (AV))

|  | **ID** | **MMV396693** | **MMV665875** | **AV** | **DA** | **CF** |
| --- | --- | --- | --- | --- | --- | --- |
| **CF** | 0.83 | 0.84 | 0.78 | 0.81 | 0.84 | 0.00 |
| **MMV665875** | 0.74 | 0.79 | 0.00 | 0.74 | 0.84 | 0.78 |
| **AV** | 0.78 | 0.83 | **0.74** | 0.00 | 0.86 | 0.81 |
| **MMV396693** | 0.78 | 0.00 | 0.79 | 0.83 | 0.81 | 0.84 |
| **ID** | 0.00 | **0.78** | **0.74** | 0.78 | 0.76 | 0.83 |
| **DA** | 0.76 | 0.81 | 0.84 | 0.86 | 0.00 | 0.84 |

**Table S3.** Primers used for determination the mRNA level of the expected target gene from *B. bovis* and *T. equi* cultures treated with MMV665875 at their IC_99_s and DMSO (0.1%) used for 8 h using a qPCR

| **Gene** | **Primer sequence** | **Parasite** | **Reference** |
| --- | --- | --- | --- |
| CP2 | Forward:5`-AGGACATTGACTGGCGTAGG-3`  Reverse: 5`-GGCAACTAACCAGCTCTTGC-3` | *B. bovis* | This study |
| CP | Forward:5`-TTGATGGAGAACCGCGTTGA-3`  Reverse: 5`- CTTCGAGAGGGATGGGAGGA-3` | *T. equi* | This study |
| *18S rRNA* | Forward:5′- GGACGCCTCGTTACTTTGAGA -3′  Reverse: 5′- AGGCGAAACCTGCTTGAAAC-3′ | *B. bovis* | (Tuvshintulga et al., 2017) |
| *18S rRNA* | Forward:5′- GCGGTGTTTCGGTGATTCATA-3′  Reverse: 5′- TGATAGGTCAGAAACTTGAATGATACATC-3′ | *T. equi* | (Kim et al., 2008) |

CP, cysteine protease gene.

**Figure S1. Anemia monitoring in *B. microti–*infected mice treated with 50 mg kg^-1^ MMV396693.** A. RBC counts. B. HGB levels. C. Hematocrit values. Each value is the mean and SD of the independent experiments. Asterisks indicate a significant difference (*P*<0.05) between treated or infected mice and uninfected mice.

**
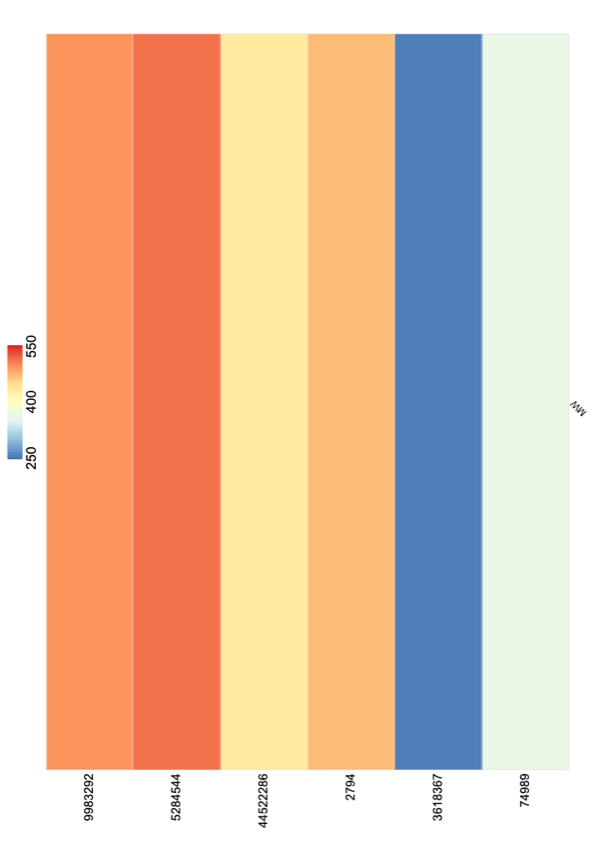
**

**Figure S2.** The molecular weight association between the powerful MMV drugs diminazene aceturate, imidocarb dipropionate, clofazimine, and atovaquone is shown in a heatmap. With Z-scores display values, a single linking mechanism was used. 3618367 = MMV396693, 44522286 = MMV665875, 5284544 = diminazene aceturate, 9983292 = imidocarb dipropionate, 2794 = clofazimine, and 74989 = atovaquone.
